# Supplementary material for: Myelination of parvalbumin interneurons shapes the function of cortical sensory inhibitory circuits
Source: Nat Commun. 2020 Oct 13;11:5151. doi: 10.1038/s41467-020-18984-7 (PMC7555533; doi:10.1038/s41467-020-18984-7)
Supplement: Supplementary file 1 — Supplementary information [file 41467_2020_18984_MOESM1_ESM.pdf]

Myelination of parvalbumin interneurons shapes the function of cortical sensory inhibitory circuits

Benamer et al.

Supplementary Information

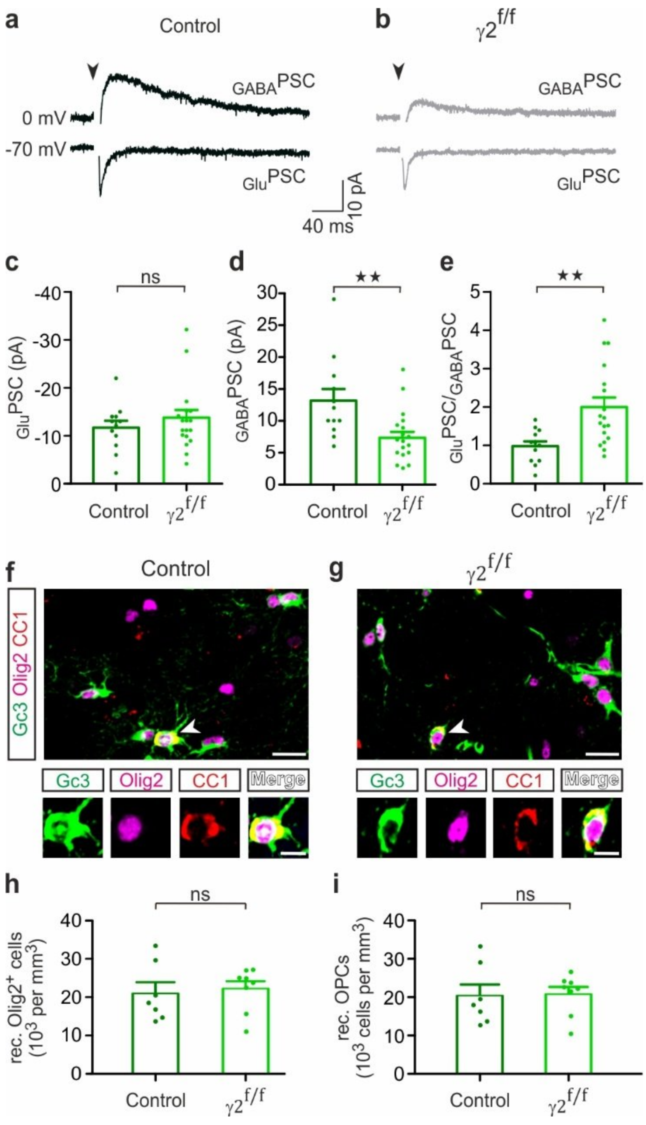

Supplementary Figure 1. Genetic and functional efficiency of the inactivation of the γ2-GABA$_A$R-mediated synapses of layer IV OPCs of the barrel cortex.

(a,b) Representative evoked glutamatergic ($_{Glu}$PSCs) and GABAergic PSCs ($_{GABA}$PSCs) in OPCs of control (**a**) and γ2$^{f/f}$ (**b**) mice in response to thalamic stimulation. Note the large decrease of $_{GABA}$PSC amplitude in the γ2$^{f/f}$ mouse. **(c,d)** Dot plots showing the amplitudes of $_{Glu}$PSCs (**c**) and $_{GABA}$PSCs (**d**) in control (dark green) and γ2$^{f/f}$ (light green) mice (n=12 and n=18 for N=3 control mice and N=4 γ2$^{f/f}$ mice, respectively; p=0.6689 and p=0.0035 for $_{Glu}$PSCs (**c**) and $_{GABA}$PSCs (**d**), two-tailed Mann–Whitney test). Stimulation artefacts were blanked for visibility. The stimulation time is indicated (arrowheads). **(e)** Dot plot of

$_{Glu}$PSC/$_{GABA}$PSC ratio obtained in OPCs of control (dark green) and $\gamma2^{f/f}$ (light green) mice (n=12 and n=18 for N=3 control mice and N=4 $\gamma2^{f/f}$ mice, respectively; p=0.0026, two-tailed Mann-Whitney U test). **(f,g)** Representative confocal images of Gcamp3 (Gc3; green), Olig2 (magenta) and CC1 (red) in a coronal section of the barrel cortex at P10 in control (**f**) and $\gamma2^{f/f}$ mice (**g**) (N= 7 mice for control mice; N=8 mice for $\gamma2^{f/f}$ mice). Note the low number of differentiated OLs at this age (solid arrowheads). Scale bars: 20 µm and 10 µm. **(h,i)** Densities of recombinant Olig2^{+} cells (**h**) and recombinant OPCs (**i**) in control (dark green) and $\gamma2^{f/f}$ mice (light green) at P10 (p=0.6943 for all recOlig2^{+} cells (**h**) and recOPC (**i**), two-tailed Mann–Whitney U test; the recombination efficiency was 65.5±3.95 %). Dot plots in **c-e, h, i** are presented as mean±s.e.m, dots in **c-e** represent data from individual recorded OPCs and dots in **h** and **i** represent data from individual mice.

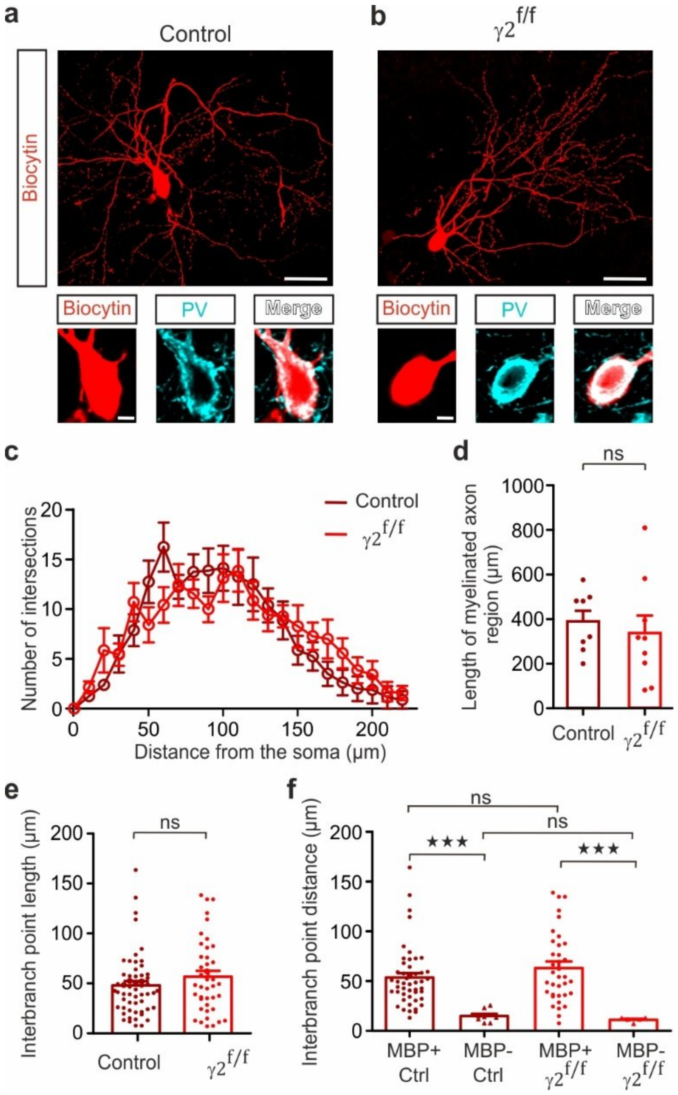

Supplementary Figure 2. Biocytin-loaded PV^{+} FSI in control and γ2$^{f/f}$ mice.

(a,b) Representative confocal images of PV^{+} FSI (cyan) loaded with biocytin (red) from control (**a**) and γ2$^{f/f}$ (**b**). Note the somatic co-localization of biocytin with PV (insets) (n=8 FSI for N=5 control mice and n=4 FSI for N=2 γ2$^{f/f}$ mice). Scale bars: 30 μm and 10 μm. **(c)** Sholl analysis of the arborization of the same reconstructed FSI axons showing no difference in the general complexity from the soma (p=0.3008, p=0.1953, p=0.7701, p=0.2810, p=0.1809, p=0.1043, p=1.000, p=0.4158, p=0.2446, p=0.8618, p=0.7719, p=0.7268, p=0.7278, p=1.0000, p=0.4855, p=0.3520, p=0.2206, p=0.1440, p=0.1565, p=0.1673, p=0.1682 and p=0.1682 from 10 μm to 220 μm, respectively; multiple two-tailed Mann-Whitney U test). **(d)**

Dot plot of the length of the myelinated axon region for the same cells. Note that this length is unchanged between control and mutants (n=8 FSI for N=5 control mice and n=9 FSI for N=3 $\gamma 2^{f/f}$ mice; p=0.4807; two-tailed Mann-Whitney U test). **(e, f)** Dot plots of all measured interbranch point lengths (**e**) and interbranch point distances including MBP^{+} versus MBP^{-} axon segments determined in the myelinated axon region (**f**) in N=5 control (dark red) and N=3 $\gamma 2^{f/f}$ (light red) mice (p=0.3443 for interbranch point length; two-tailed Mann–Whitney U test; p=0.0005 and p=0.0003 between MBP^{+} and MBP^{-} in control and $\gamma 2^{f/f}$ mice, respectively, and p=0.9692 and p=1 for MBP^{+} in control and $\gamma 2^{f/f}$ mice and for MBP^{-} in control and $\gamma 2^{f/f}$ mice, respectively; one-way Kruskal-Wallis test followed by a Bonferroni's multiple comparison post hoc test). Dot plots in **d**, **e** and **f** are presented as mean±s.e.m, dots in **d** represent data from individual FSI and dots in **e** and **f** represent all interbranch segments of analyzed FSI.

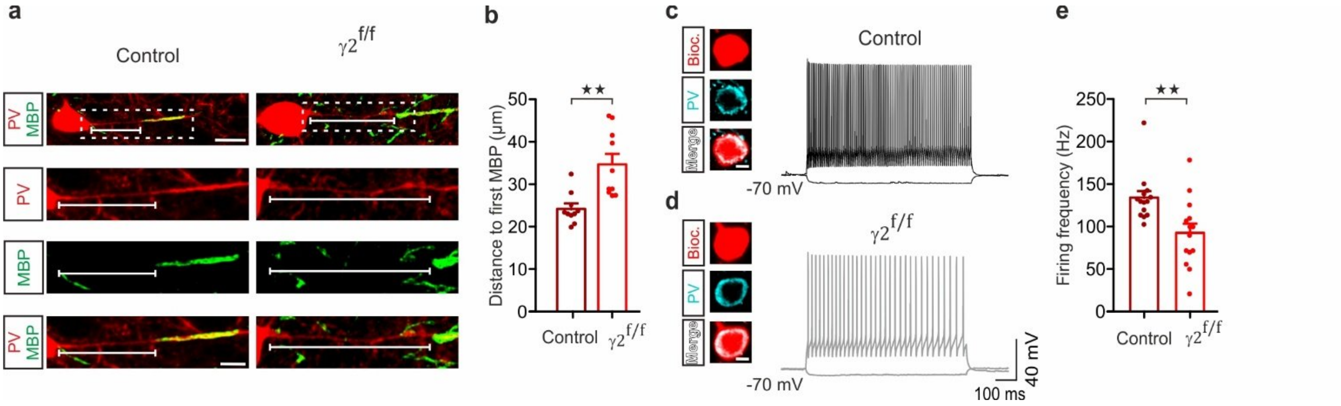

Supplementary Figure 3. Myelination and firing frequency of PV^+ FSI in layer V of the barrel cortex.

(**a**) Confocal images of layer V FSI of the barrel cortex showing a PV^+ axon (red) colocalized with MBP (green) in control (left) and $\gamma 2^{f/f}$ (right) mice. Note that the distance from the soma to the onset of MBP signal is longer in the $\gamma 2^{f/f}$ mouse compared to the control (lines). Scale bars: 15 µm and 10 µm. **(b)** Dot plot of the distances from the soma to the first MBP signal in control (dark red) and $\gamma 2^{f/f}$ (light red) mice (n=10 for N=6 control and N=5 $\gamma 2^{f/f}$ mice; p=0.0013, two-tailed Mann–Whitney U test). **(c,d)** Current clamp recordings of FSI held at -70 mV during injections of 200 pA and -50 pA in control (**c**) and $\gamma 2^{f/f}$ mice (**d**). Note the PV expression in both recorded FSI (insets) and the decreased firing frequency in the mutant. (**e**) Dot plots of the frequency of action potential discharges in control (dark red) and $\gamma 2^{f/f}$ mice (n=14 for N=13 control and n=14 for N=10 $\gamma 2^{f/f}$ mice p=0.0024; two-tailed Mann-Whitney U test). Dot plots in **b** and **e** are presented as mean±s.e.m and dots represent data from individual FSI.

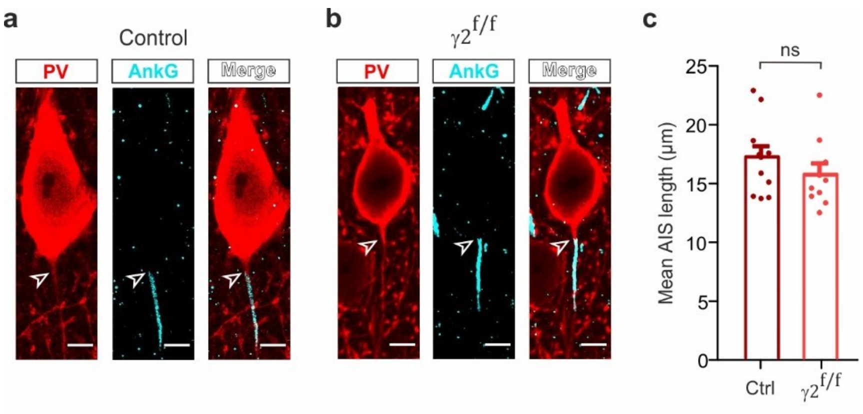

Supplementary Figure 4. Similar AIS length in control and γ2$^{f/f}$ mice.

(a,b) Confocal images of layer IV FSI of the barrel cortex showing a PV^{+} axon (red, open arrowhead) colocalized with the AIS marker Ankyrin G (cyan) in control (**a**) and γ2$^{f/f}$ (**b**) mice. Note that they have a similar length and arise from the same distance from the soma (open arrowhead; length to Ankyrin G: 2.84±0.33 µm and 3.02±0.45 µm for n=10 cells in N=2 control and n=9 in N=2 γ2$^{f/f}$ mice, respectively; p=0.7569, two-tailed Student t test). Scale bar: 5 µm. **(c)** Dot plot of the AIS length in control (dark red) and γ2$^{f/f}$ (light red) mice (n=10 cells for N=2 control and N=2 γ2$^{f/f}$ mice, respectively; p=0.4, two-tailed Student t test).

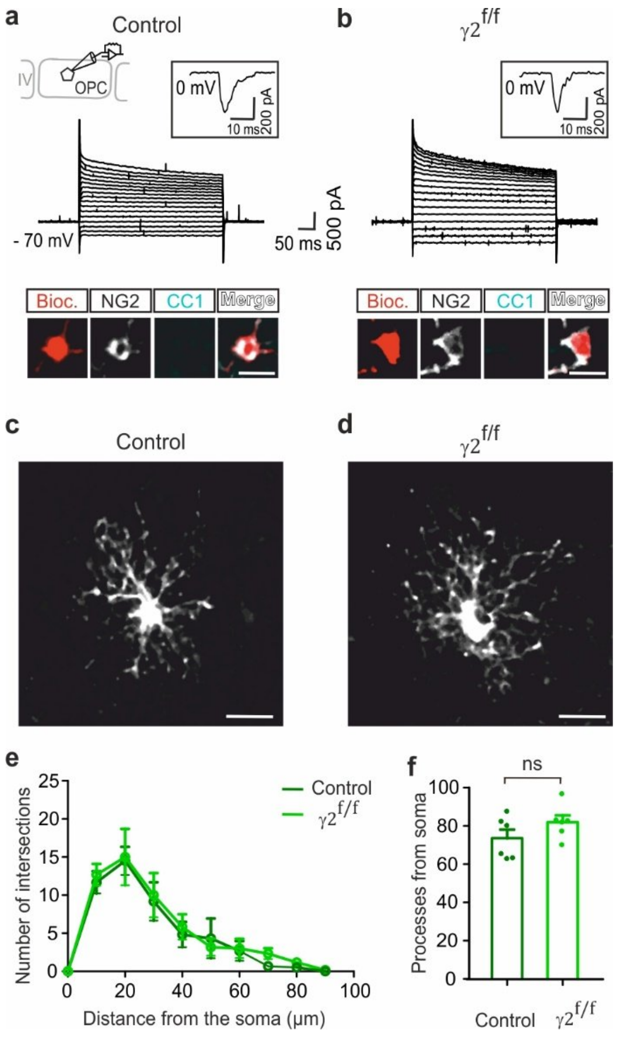

Supplementary Figure 5. Recombinant layer IV OPCs exhibited a similar morphological complexity in control and γ2$^{f/f}$ mice.

(a,b) Currents induced by voltage steps from +60 mV to −100 mV for biocytin-loaded recombinant cells held at −70 mV, positive for the OPC maker NG2 and negative for the OL marker CC1 from control (**a**) and γ2$^{f/f}$ (**b**) mice. Note the rectifying I-V curve and the presence of a Na^{+} current (top insets) of these cells in both groups. Scale bar: 10 µm. **(c,d)** Representative confocal images of recorded recombinant layer IV OPCs loaded with biocytin (gray) from control (**c**) and γ2$^{f/f}$ (**d**) mice (n=6 cells from N=3 control and N=3 γ2$^{f/f}$ mice, respectively). Scale bar: 40 µm. **(e)** Sholl analysis of the arborization of the same recorded recombinant layer IV OPCs that appear equivalent in control and γ2$^{f/f}$ mice (n=6 cells from N=3 control and N=3 γ2$^{f/f}$ mice, respectively; p=0.7479, p=1.0000, p=0.9372, p=0.6831,

p=0.9318 and p=0.7888 at 10 µm, 20µm, 30 µm, 40 µm, 50 µm and 60 µm, respectively; multiple two-tailed Mann-Whitney U test). **(f)** Dot plots of the number of branches from soma per OPC in control (dark green) and $\gamma 2^{f/f}$ (light green) mice (p=0.2607, two-tailed Mann-Whitney U test). Data in **e** and **f** are presented as mean±s.e.m and dots in **f** represent data from individual OPCs.

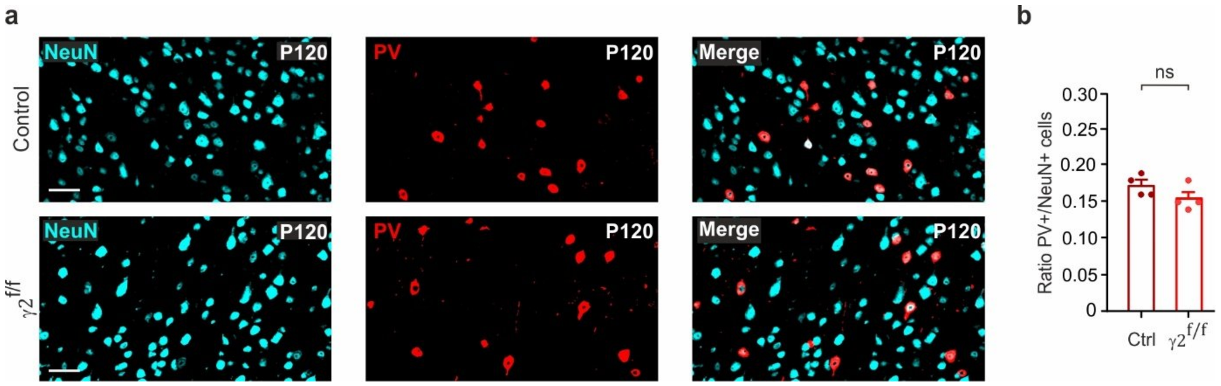

Supplementary Figure 6. Normal PV expression in control and γ2$^{f/f}$ mice at P120.

(a) Representative confocal images of NeuN^{+} neurons (Cyan) and PV^{+} interneurons (red) in control (top) and γ2$^{f/f}$ mice (bottom) at P120. Scale bar: 40 μm. (**b**) Dot plot of the ratio of PV^{+} cells/NeuN^{+} cells in control (dark red) and γ2$^{f/f}$ mice (light red) at P120 (N=4 mice per condition; p=0.1775, unpaired two-tailed Student t test). Dot plot in **b** is presented as mean±s.e.m and represents data for individual mice.

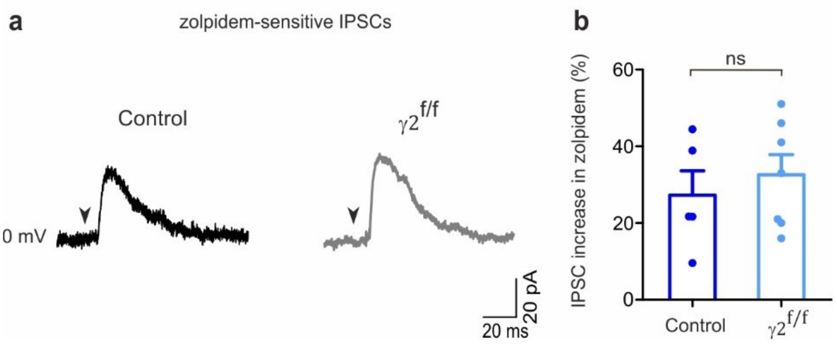

Supplementary Figure 7. Postsynaptic γ2-GABA$_A$R in SSCs are functional in control and γ2$^{f/f}$ mice.

(a) Zolpidem-sensitive IPSCs recorded in layer IV SSCs of the barrel cortex of controls (black) and γ2$^{f/f}$ mice (gray). These IPSCs were obtained by subtracting mean IPSCs in zolpidem (1µM) from mean IPSCs before drug application. (**b**) Dot plots showing the similar effect of zolpidem (1 µM) in SSCs from control (dark blue, n=5 from N=2 mice) and γ2$^{f/f}$ mice (light blue, n=7 from N=3 mice; p=0.7551, two-tailed Mann-Whitney U test). Dot plots are presented as mean±s.e.m, dots represent data from individual recorded SSCs.

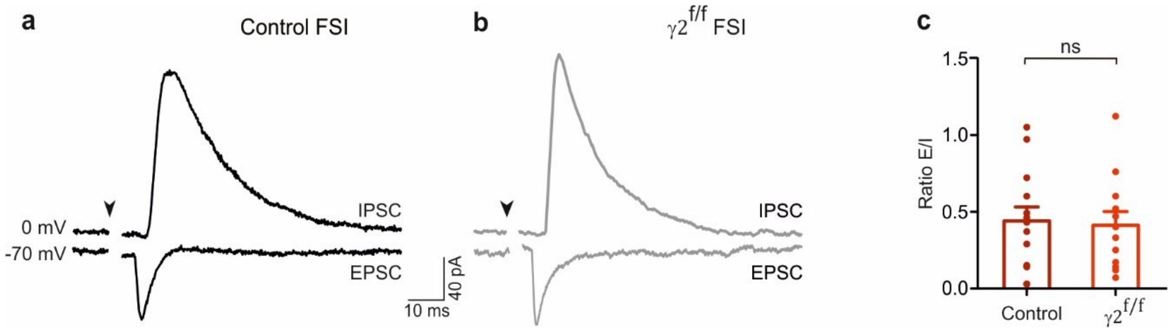

Supplementary Figure 8. Thalamocortical feedforward inhibition in postsynaptic layer IV FSI in control and γ2$^{f/f}$ mice.

(a,b) Evoked EPSCs (bottom trace) and IPSCs (top trace) in layer IV FSI held at -70 mV and -0 mV, respectively, upon thalamic stimulation in control (black, P27) and γ2$^{f/f}$ (gray, P25) mice. Stimulation artefacts were blanked for visibility. The stimulation time is indicated (arrowheads). **(c)** Dot plots showing no significant difference in E/I ratio in FSI of control (dark red, n=13 from N=3 mice) and γ2$^{f/f}$ mice (light red, n=12 from N=3 mice; p=0.8267, two-tailed Student's t test). Dot plots are presented as mean±s.e.m and dots represent data from individual recorded FSI.

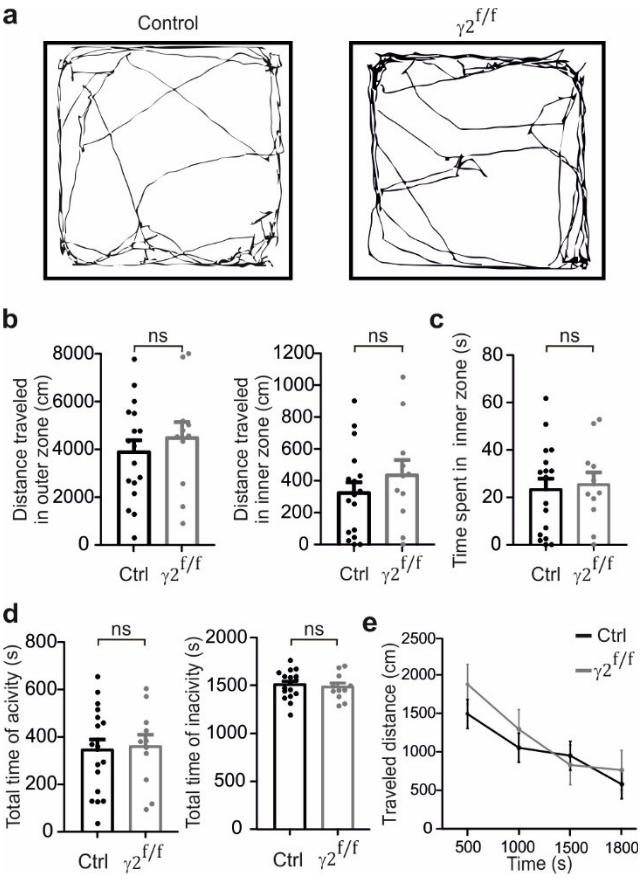

Supplementary Figure 9. Open-field behavior in control and γ2$^{f/f}$ mice.

(a) Open field trajectory maps during 3 min in control (left) and γ2$^{f/f}$ (right) mice. **(b,c,d)** Dot plots of total distance traveled in the outer and inner zones (**b**), time spent in inner zone (**c**) and total time of activity and inactivity (**d**) in N=17 control and N=11 γ2$^{f/f}$ mice (p=0.479 and p=0.337 for **b**, p=0.767 for **c,** and p=0.825 and p=0.653 for **d**; two-tailed Student's t test. **(e)** Time course of the traveled distance (N=17 control and N=11 γ2$^{f/f}$ mice). Data in **b-e** are presented as mean±s.e.m and dots in **b-d** represent data from individual mice.

Supplementary Table 1: Electrophysiological properties of FSI and SSCs in control and γ$2^{f/f}$ mice

Parameter	Ctrl FSI (n=26)	γ$2^{f/f}$ FSI (n=28)	p
Rin (MΩ)	120.92 ± 7.97	127.17 ± 15.17	p=0.1643
Em (mV)	-63.38 ± 0.98	-61.78 ± 1.09	p=0.2980
Threshold (mV)	-44.66 ± 1.19	-47.08 ± 0.97	p=0.2771
1st spike duration (ms)	0.72 ± 0.05	0.72 ± 0.06	P=0.9456
2nd spike duration (ms)	0.76 ± 0.05	0.74 ± 0.06	p=0.8144
1st spike amplitude (mV)	70.76 ± 2.12	70.92 ± 3.37	p=0.5949
2nd spike amplitude (mV)	67.77 ± 2.04	68.17 ± 3.17	p=0.8993
AHP (mV)	-12.47 ± 1.87	-13.50 ± 0.84	p=0.6433
F_{max} (Hz)	140.31 ± 10.19	103.54 ± 6.14	p=0.0108
	Ctrl SSCs (n=18)	γ$2^{f/f}$ SSCs (n=18)	
Rin (MΩ)	220.56 ± 24.87	173.52 ± 16.29	p=0.1446
Em (mV)	-62.82 ± 1.03	-65.13 ± 1.19	p=0.6192
Treshold (mV)	-40.66 ± 0.95	-43.05 ± 0.84	p=0.0772
1st spike duration (ms)	1.97 ± 0.13	1.71 ± 0.09	p=0.1647
2nd spike duration (ms)	2.34 ± 0.11	2.03 ± 0.12	p=0.0518
1st spike amplitude (mV)	81.23 ± 3.05	75.66 ± 2.91	p=0.3934
2nd spike amplitude (mV)	75.64 ± 3.25	70.25 ± 3.11	p=0.3509
AHP (mV)	-2.55 ± 0.94	-4.51 ± 1.45	p=0.3308
F_{max} (Hz)	31.67 ± 1.51	33.44 ± 1.92	p=0.3987
	Ctrl pyramidal cell (n=9 ; N= 2 mice)	γ$2^{f/f}$ pyramidal cell (n=11 ; N=2 mice)	
Rin (MΩ)	261.10 ± 48.98	218.00 ± 36.18	p=0.8197
Em (mV)	-70.89 ± 2.15	-70.82 ± 1.00	p=0.8491
Treshold (mV)	-55.89 ± 1.20	-54.30 ± 1.58	p=0.4033
1st spike duration (ms)	2.02 ± 1.40	1.99 ± 1.15	p=0.6758
2nd spike duration (ms)	2.65 ± 0.20	2.57 ± 0.16	p=0.9197
1st spike amplitude (mV)	79.04 ± 3.46	76.48 ± 4.81	p=0.7612
2nd spike amplitude (mV)	74.34 ± 3.39	72.02 ± 4.61	p=0.9697
AHP (mV)	-0.94 ± 1.25	-1.18 ± 0.55	p=0.8792
F_{max} (Hz)	30.67 ± 3.25	31.27 ± 2.64	p=0.8789

The characterization of FSI and SSC electrical properties in control and γ$2^{f/f}$ mice was done as previously described[14]. The input resistance (Rin) and membrane potential (Em) were measured in current clamp by applying hyperpolarizing pulses of -50 pA from −70 mV. We dissected the spike morphology from action potentials elicited by 80 ms suprathreshold pulses from −70 mV (600 pA). From these recordings, the spike threshold corresponded to the voltage at which the derivative of the action potential (dV/dt) experienced a twofold increase.

The amplitudes of the first and the second action potential, were calculated from the threshold to peak. Their duration corresponded to the full-width at half maximum (FWHM) from a Gaussian fit of the depolarized face of the action potential immediately after the threshold. Finally, the amplitude of AHP was calculated as the difference between the threshold and the peak of the fast after-hyperpolarization. The maximal firing frequency was determined during suprathreshold pulses in current clamp configuration from −70 mV (200 pA, 900 ms) and calculated as the number of spikes divided by the pulse duration (Fmax) (two-tailed Student t test for FSI and two-tailed Mann Whitney U test for SSCs and pyramidal cells). Values are presented as mean±s.e.m.

Supplementary Table 2: Morphological and electrophysiological parameters of the model

Parameter	Value	Units
Morphology		
Number of internodes	15[9]	
Number of nodes	16	
Internode diameter	0.58[7]	µm
Node diameter	0.54[7]	µm
G ratio	0.66[7]	
Myelin thickness	0.1326[7]	µm
Number of myelin lamellae	9	
Myelin lamella periodicity	15.6	nm
Periaxonal space width	15	nm
Electrophysiology		
Leakage potential/Node leak reversal potential	-83.3814[21]	mV
Na+ reversal potential	50	mV
K+ reversal potential	-84	mV
Node leak conductance	Adjusted to set Vm	mS/mm²
Internode leak conductance	0.1[21]	mS/cm²
Myelin membrane conductance	1[21]	mS/cm²
Node capacitance	0.9[21]	µF/cm²
Internode capacitance	0.9[21]	µF/cm²
Myelin membrane capacitance	0.9[21]	µF/cm²
Axoplasmic resistivity	0.7[21]	Ω*m
Periaxonal resistivity	0.7[21]	Ω *m
Fast Na+ conductance	30[21]	mS/mm²
Persistent Na+ conductance	0.05[21]	mS/mm²
Na+ reversal potential	50[21]	mV
Slow K+ conductance	0.8[21]	mS/mm²
K+ reversal potential	-84[21]	mV
