# Supplementary material for: Myelination of parvalbumin interneurons shapes the function of cortical sensory inhibitory circuits
Source: Nat Commun. 2020 Oct 13;11:5151. doi: 10.1038/s41467-020-18984-7 (PMC7555533; doi:10.1038/s41467-020-18984-7)
Supplement: Supplementary file 3 — Reporting Summary [file 41467_2020_18984_MOESM3_ESM.pdf]

nature research

Corresponding author(s): Maria Cecilia Angulo

Last updated by author(s): Sep 10, 2020

Reporting Summary

Nature Research wishes to improve the reproducibility of the work that we publish. This form provides structure for consistency and transparency in reporting. For further information on Nature Research policies, see Authors & Referees and the Editorial Policy Checklist .

Statistics

For all statistical analyses, confirm that the following items are present in the figure legend, table legend, main text, or Methods section.

n/a	Confirmed	
[ ]	[x]	The exact sample size (*n*) for each experimental group/condition, given as a discrete number and unit of measurement
[ ]	[x]	A statement on whether measurements were taken from distinct samples or whether the same sample was measured repeatedly
[ ]	[x]	The statistical test(s) used AND whether they are one- or two-sided<br>*Only common tests should be described solely by name; describe more complex techniques in the Methods section.*
[x]	[ ]	A description of all covariates tested
[ ]	[x]	A description of any assumptions or corrections, such as tests of normality and adjustment for multiple comparisons
[ ]	[x]	A full description of the statistical parameters including central tendency (e.g. means) or other basic estimates (e.g. regression coefficient) AND variation (e.g. standard deviation) or associated estimates of uncertainty (e.g. confidence intervals)
[ ]	[x]	For null hypothesis testing, the test statistic (e.g. *F*, *t*, *r*) with confidence intervals, effect sizes, degrees of freedom and *P* value noted<br>*Give P values as exact values whenever suitable.*
[x]	[ ]	For Bayesian analysis, information on the choice of priors and Markov chain Monte Carlo settings
[x]	[ ]	For hierarchical and complex designs, identification of the appropriate level for tests and full reporting of outcomes
[ ]	[x]	Estimates of effect sizes (e.g. Cohen's *d*, Pearson's *r*), indicating how they were calculated

Our web collection on statistics for biologists contains articles on many of the points above.

Software and code

Policy information about availability of computer code

Data collection	Cells were visualized under the microscope with Imaging Workbench version 6.0 software. Acquistion electrophysiological data was obtained using pClamp10.3 software. Confocal images were acquired with LAS X software (version 3.7.2.22383) .
Data analysis	Electrophysiology was analyzed with pClamp10.5 software and Neuromatic package within IGOR Pro 6.0 environment and confocal images with NIH ImageJ software (version 1.52i). Conduction velocity was simulated with Matlab (version: R2018b). Open field data were automatically processed with ViewPoint software (version 2011). Statistically analysis were done with GraphPad Prism (version 5 and 8).

For manuscripts utilizing custom algorithms or software that are central to the research but not yet described in published literature, software must be made available to editors/reviewers. We strongly encourage code deposition in a community repository (e.g. GitHub). See the Nature Research guidelines for submitting code & software for further information.

Data

Policy information about availability of data

All manuscripts must include a data availability statement. This statement should provide the following information, where applicable:
- Accession codes, unique identifiers, or web links for publicly available datasets
- A list of figures that have associated raw data
- A description of any restrictions on data availability

The source data underlying all Figures, Supplementary Figures, and Table are provided as a Source Data file with the paper.

Field-specific reporting

Please select the one below that is the best fit for your research. If you are not sure, read the appropriate sections before making your selection.

Life sciences study design

All studies must disclose on these points even when the disclosure is negative.

Sample size	No specifical tests were used to determine sample size. Samples sizes were chosen based on available information on variability from previous electrophysiological, behavioural and immunohistochemical datasets (see Balia et al., (2017) Glia; Orduz et al., (2019) Nat. Commun).
Data exclusions	Biocytin-loaded FSI were excluded of morphological reconstructions when the cells were faintly labelled or not found after immunostainings . Electrophysiological data were excluded from the analysis when the series resistance of the cells changed of more than 30%. Only an exceptional behavioral data point submitted to Grubbs test was excluded as an extreme outlier.
Replication	All attempts of replication were successful. Experiments were replicated at least 3 times with different animals to verify reproducibility.
Randomization	We did not randomize the data since experiments depended on the genotype of each mouse. However, we made sure that we have a relatively equal n size per condition for each set of experiment.
Blinding	Blinding was not possible because both control and mutant mice required a tamoxifen-induced protocol and thus were recognized by the experimenter..

Reporting for specific materials, systems and methods

We require information from authors about some types of materials, experimental systems and methods used in many studies. Here, indicate whether each material, system or method listed is relevant to your study. If you are not sure if a list item applies to your research, read the appropriate section before selecting a response.

Materials & experimental systems

n/a	Involved in the study	
☐	☒	Antibodies
☒	☐	Eukaryotic cell lines
☒	☐	Palaeontology
☐	☒	Animals and other organisms
☒	☐	Human research participants
☒	☐	Clinical data

Methods

n/a	Involved in the study	
☒	☐	ChIP-seq
☒	☐	Flow cytometry
☒	☐	MRI-based neuroimaging

Antibodies

Antibodies used	Primary antibodies used for immunohistochemistry were rabbit polyclonal anti-Olig2 (1:400; ref. AB9610, Millipore), chicken polyclonal anti-GFP (1:1000; ref. A10262, ThermoFisher Scientific), rabbit polyclonal anti-PV (1:1000; ref. PV-27, Swant), mouse monoclonal anti-APC (CC1; (1:100; clone CC-1; ref. OP80, Millipore), rabbit polyclonal anti-NG2 (1:100; ref. AB5320, Thermofisher Scientific), rat monoclonal anti-MBP (1:100; clone 26; ref. AB7349, Abcam), mouse monoclonal anti-NeuN (1:250; clone A60; ref. MAB377, Merck), rmouse monoclonal anti-Ankyrin G (1:100; clone N106/36; ref. MAB1683, Merck) and rabbit polyclonal anti-Caspr (1:500; ref. ab34151, Abcam).<br>Secondary polyclonal antibodies were goat anti-rabbit DyLight-405 (ref. 35551; A-11039, Thermofisher Scientific), goat anti-chicken Alexa Fluor-488 (ref. A-11039, Thermofisher Scientific), goat anti-mouse Alexa Fluor-546 (ref. A-11030, Thermofisher Scientific), goat anti-mouse DyLight-633 (ref. GTX76787; Genetex) or goat anti-rat Alexa Fluor-633 (ref. A-21094, Thermofisher Scientific). We also used the conjugated streptavidin–Alexa Fluor-546 (1:200; ref. S11225) to reveal biocytin.
Validation	All primary antibodies have been validated for immunohistochemistry and western blots for use on mouse. More detailed information, including citation, can be found in manifacturer websites. Tissue immunostainings omitting primary anitbodies were used to rule out the possibility of non-specific binding of secondary antibodies.

Animals and other organisms

Policy information about studies involving animals; ARRIVE guidelines recommended for reporting animal research

Laboratory animals	Mus musculus (mouse), various transgenic lines with C57Bl/6 genetic background, ages from postnatal day 10 to postnatal day 120, both sex.
Wild animals	No wild animals were used in this study.
Field-collected samples	No samples were collected from the field.
Ethics oversight	All experiments of the present study followed European Union and institutional guidelines for the care and the use of laboratory animales and were approved by the French ethical committee for animal care of the University Paris Descartes (Committee N°

CEEA34) and the Ministry of National Education and Research (Project N°: 13094-2017081712355709).

Note that full information on the approval of the study protocol must also be provided in the manuscript.
